# Chronic Endotoxemia in Subjects with Type-1 Diabetes Is Seen Much before the Onset of Microvascular Complications

**DOI:** 10.1371/journal.pone.0137618

**Published:** 2015-09-14

**Authors:** Vivekanandhan Aravindhan, Viswanathan Mohan, Namasivayam Arunkumar, Sreedharan Sandhya, Subash Babu

**Affiliations:** 1 AU-KBC Research Centre, MIT Campus of Anna University, Chennai, India; 2 Dept of Genetics, Dr ALM PG IBMS, University of Madras, Taramani, Chennai, 600113, India; 3 Madras Diabetes Research Foundation & Dr. Mohan’s Diabetes Specialties Centre, WHO Collaborating Centre for Non-Communicable Diseases Prevention and NGT, International Diabetes Federation (IDF) Centre for Education, Chennai, India; 4 Biostatistics & Programming, Algorithm Inc, Bengaluru, India; 5 National Institutes of Health-International Center for Excellence in Research, National Institute for Research in Tuberculosis, Chennai, India; Queen's University Belfast, UNITED KINGDOM

## Abstract

**Background:**

Lipopolysaccharide (LPS)/Endotoxin is hypothesized to play an important role in chronic inflammation associated with Type-1 diabetes (T1DM) and its complications. Endotoxin core antibodies (EndoCAb), LPS binding protein (LBP) and soluble CD14 (sCD14) act as modulators of LPS induced activation of innate immune system *in vivo*. For the present study we estimated the levels of LPS and its translocation markers in T1DM subjects with and without microvascular complications (MVC) and correlate them with clinical parameters of T1DM and serum inflammatory cytokine levels (TNF-α, IL-6, IL-1β and GM-CSF).

**Methods:**

A total of 197 subjects (64 normal glucose tolerance (NGT) subjects, 97 T1DM subjects without MVC and 36 with MVC) were included in this study and the levels of serum LPS, its translocation markers and cytokines measured by immunoassays.

**Results:**

Compared to NGT, T1DM subjects (both with and without MVC) had significantly higher levels of LPS, reduced levels of LBP and EndoCAb along with significant increase in the levels of IL-1β, IL-6, TNF-α and GM-CSF (p<0.05). No significant change was seen in the levels of these biomarkers between T1DM subjects with and without MVC.

**Conclusions:**

Decreased levels of EndoCAb and LBP suggest sustained endotoxin activity in T1DM subjects even before the onset of microvascular complications.

## Background

Type-1 diabetes mellitus (T1DM) is a bonafide autoimmune disorder characterized by chronic inflammation with rapid loss of pancreatic beta cells leading to insulin deficiency [1]. However, the exact cause of autoimmunity is not known, even though, both genetic [2] and environmental factors have been implicated [3]. The prevalence of T1DM and other autoimmune diseases has increased dramatically over the past few decades [4]. Although genetic factors may play an important role in the susceptibility to T1DM, the dramatic worldwide increase in prevalence is likely due to changes in the environmental factors [5]. One environmental factor which has gained prominence in recent years is the alterations in gut microbiota which leads to increased gut permeability and metabolic endotoxemia [6]. Bacterial Lipopolysaccaride (LPS)/ endotoxin, a unique glycolipid located at the outer membrane of gram negative bacteria can induce inflammation [7]. More than the actual endotoxin levels, the levels of endogenous anti-endotoxin core antibodies (EndoCAb), LPS binding protein (LBP) and soluble CD14 (sCD14) were found to be more important in determining the activity of endotoxin under *in vivo* conditions [8]. Studies reporting levels of EndoCAb, LBP and sCD14 in T1DM subjects are not available. While EndoCAb antibodies can neutralize LPS, sCD14 and LPB can augment/antagonize LPS activity in a dose dependent manner. Recent studies have demonstrated chronic inflammation in T1DM subjects with microvascular complications (MVC) with systemic increase in the levels of pro-inflammatory cytokines [9]. We hypothesized that a systemic increase in endotoxemia could aggravate inflammation and promote MVC. The objective of the present study was to estimate the levels of LPS and its translocation markers in T1DM subjects with/without MVC (DN and/or DR) and correlate them with clinical parameters for T1DM and serum inflammatory cytokines (TNF-α, IL-6, IL-1β and GM-CSF).

## Materials and Methods

### Study participants

Patients with T1DM (n = 133; 97 T1DM without MVC and 36 T1DM with MVC) were recruited from Dr. Mohan’s Diabetes Specialties Centre, Chennai, India. T1DM was diagnosed by the absence of insulin reserve shown by C-peptide assay (C-peptide values < 0.3 pg/ml) and requiring insulin from the time of diagnosis. Subjects with serum glutamic acid decarboxylase (GAD)–specific autoantibody levels ≥ 10 IU/ml were classified as GAD^+^. Only fasting blood samples were used for all analysis. Institutional Ethical Committee approval was obtained from the Madras Diabetes Research Foundation Ethics Committee (Ref. No.MDRF-EC/SOC/2009/05), and written informed consent was obtained from all the study participants. The study was conducted as per principles of the declaration of Helsinki as revised in 2008.

### Study Design and sample size calculation

It is a cross-sectional observational study. Initially, 20 normal glucose tolerant (NGT) and 20 age and gender matched T1DM subjects with and without MVC were used for analysis. On the basis of the preliminary results, with a confidence interval of 95%, an estimated p value < 0.05, and a power of 80%, the sample size was estimated to be 150 ie, 60 NGT subjects, 60 T1DM subjects without MVC and 30 T1DM with MVC. Few more samples were included to account for the large variation seen in serum biomarker levels.

### Estimation of biochemical parameters

Blood parameters were measured using a Hitachi-912 Autoanalyser (Hitachi, Mannheim, Germany). Glycated hemoglobin (HbA1c) was estimated by high pressure liquid chromatography (Bio-Rad, Hercules, CA). Urine samples were collected in the early morning after an overnight fast. Urine creatinine was measured using Jaffe’s method. Urine microalbumin concentration was measured using commercially available immunoturbidometric assay kits from Randox (Randox, UK) on Opera Technicon Auto Analyser (Bayer Diagnostics, USA). The urine sample was added to a buffer containing anti-albumin antibody. The turbidity of the resulting solution was measured and the albumin concentration was determined by constructing a standard curve with known concentrations of albumin. The mean inter-assay and intra-assay coefficient of variation were 3.4% and 2.4% respectively. Microalbuminuria was diagnosed if the albumin excretion was between 30 and 299 μg/mg of albumin [10]. The expected protein excretion (EPE) was calculated as the urinary protein to creatinine ratio [11]. The intra- and inter assay coefficient of variation for the biochemical assays ranged between 3.1% and 5.6%.

### Screening for microvascular and macrovascular complications

All T1DM subjects were screened for both microvascular (diabetic retinopathy/DR, diabetic nephropathy/DN and diabetic neuropathy) and macrovascular complications (diabetic coronary artery disease/ DM-CAD and perivascular diseases/DM-PVD).

#### Doppler screening

Doppler screening for PVD was performed by recording of pressure tracings while in the supine position by doppler probe using the KODY Vaslab Machine (Kody Labs, Chennai, India). The ankle–brachial index (ABI) ratio was calculated in every subject as previously described [12].

#### Retinal photography

Screening for retinopathy was done using four-field stereo colour retinal photography (Zeiss FF 450 plus camera) which were graded by an ophthalmologist using the Early Treatment Diabetic Retinopathy Study (ETDRS) grading system as previously described [13].

#### Biothesiometry studies

Biothesiometer (Biomedical Instrument Co., Newbury, OH, USA) was used to assess vibratory perception threshold (VPT) of the great toes in a standardized fashion as previously described [12].

#### Electrocardiogram

Resting 12-lead electrocardiogram (ECG) was performed using Myocard R electrocardiograph (Marks Electronics, Chennai, India) to asses CAD. Carotid Intimal Medial Thickness (IMT) was measured as previously described [13].

#### Arterial stiffness measurement

Arterial stiffness was measured using the Sphygmocor apparatus (Sphygmocor BPAS-1; PWV Medical, Sydney, Australia). Augmentation index was defined as the difference between the first and second peaks of the central arterial waveform, expressed as a percentage of the pulse pressure as previously described [13].

### Definitions and cutoffs

#### Hypertension

It was diagnosed in subjects who were on antihypertensive medication or had systolic BP≥140 mmHg or diastolic BP ≥90 mmHg as previously described [12, 13].

#### Diabetic Perivascular disease

An ABI of ≤0.9 was the criterion used for the diagnosis of PVD as per American College of Cardiology Foundation/American Heart Association Task Force (ACCF/AHA) 2011 Guidelines as previously described [12, 13].

#### Diabetic Coronary Artery Disease

This was diagnosed based on a past history of documented myocardial infarction and/or drug treatment for CAD (aspirin or nitrates) and/or electrocardiographic changes suggestive of ST segment depression and/or Q-wave changes and/or T-wave changes using appropriate Minnesota codes as previously described [12, 13].

#### Diabetic Retinopathy

The minimum criteria for diagnosis of DR were the presence of at least one definite microaneurysm in any field photographed as previously described [12, 13].

#### Diabetic Neuropathy

Diagnosed if VPT of the great toe exceeded mean + 2 SD of a healthy nondiabetic population aged 20–45 years (cut point ≥20 V) as previously described [12, 13].

#### Diabetic Nephropathy

Microalbuminuria was diagnosed if the albumin excretion was between 30 and 299 μg/mg of creatinine and macroalbuminuria/overt nephropathy was diagnosed if albumin excretion was ≥300 μg/mg of creatinine as previously described [12, 13].

### Inclusion and exclusion criteria

Only adult type-1 diabetic subjects (age 30–50) were included in the study. The exclusion criteria were patients with type-2 diabetes and T1DM subjects with macrovascular complications including cardiovascular disease and perivascular disease. Patients with a previous diagnosis of urolithiasis or any known renal disease, liver cirrhosis, congestive heart failure, chronic lung diseases, chronic infections and viral hepatitis were also excluded.

### Detection of serum LPS and translocation markers

Serum lipopolysaccaride (LPS) activity was determined with a Limulus amoebocyte lysate assay (Hycult Biotechnology). Serum LBP (Hycult Biotechnology), IgG antibody to LPS core (EndoCAb) (Hycult Biotechnology) and sCD14 (R&D Systems, Abingdon, United Kingdom) levels were measured by ELISA. The detection limit for LPS, LBP, sCD14 and EndoCAb were 0.04 Endotoxin Units (EU) /ml, 4.4 ng/ml, 0.125ng/ml and 0.125 EndoCAb Median Unit (EMU)/ml respectively. One EU equals approximately 0.1 to 0.2ng endotoxin/mL.

### Estimation of serum pro-inflammatory cytokines

Serum levels of IL-1beta, IL-6, TNF-alpha and GM-CSF were determined by ELISA following kit protocol (Invitrogen CytoSet^TM^ for IL-1β, IL-6 and TNF-α; R&D DuoSet Antibody pair for GM-CSF). The lowest detection limits were: TNF-α-1.0 pg/ml, IL-6- 0.1 pg/ml, IL-1β -0.01 pg/ml and GM-CSF-0.01pg/ml.

### Statistical analysis

Student t-test was used to compare groups for continuous variables, whereas χ2 test or Fisher exact test (as appropriate) was used to compare proportions. Mann–Whitney U test was used for multiple pair-wise comparisons that did not show normal distribution. Multiple comparisons were corrected using the Holm’s correction. Spearman’s Correlation analysis was performed between serum inflammatory markers and clinical parameters with the diabetic group. Since there was no statistical difference in the levels of inflammatory markers between the T1DM and T1DM-MVC groups, both groups were pooled for the correlation analysis. All the analyses were done using GraphPad Prism version 5.0 (GraphPad Software, CA, USA) and SPSS statistical package (Version 20.0; SPSS, Chicago, IL). p value less than 0.05 was considered significant.

## Results and Discussion

Table 1 shows the clinical and biochemical characteristics of the study subjects. Compared with the NGTs, T1DM subjects (both with/without MVC) had significantly higher levels of FPG and EPE and lower levels of total cholesterol and LDL cholesterol. In general, the serum cholesterol levels as seen in this study (total, LDL and HDL), were 2–3 times higher compared to those values reported in the Caucasian population. This is due to genetic susceptibility of the Asian Indians to hypercholestremiea which is part of the “Asian Indian” or “south-Asian” phenotype. Systolic BP, diastolic BP, serum urea and microalbuminuria were significantly elevated only in the T1DM-MVC group. 3% of T1DM and 5% (T1DM-MVC) had hypertension and were under hypertensive drugs. 37% of T1DM and 28% T1DM-MVC were GAD antibody positive. All the T1DM subjects had well established disease with minimal pancreatic beta cell reserve (as determined by C-peptide assay) and were under insulin. Among subjects with MVC, 42% had diabetic retinopathy (DR), 44% had diabetic nephropathy (DN) and 14% had both DN and DR.

**Table 1:** Clinical characteristics of the study population.

Parameters	NGT (n = 64)	T1DM without MVC^a^ (n = 97)	T1DM with MVC^b^ (n = 36)
**Gender (male/female)**	32/32	49/48	19/17
**Age [Years]**	30.40 ± 15.76	29.02 ± 16.94	34.47 ± 16.03
**Body mass index [kg/m** ^**2**^ **]**	20.19 ± 4.07	19.66 ± 5.60	20.74 ± 4.3
**FPG (mmol/L)**	4.72 ±0.33	**11.3 ± 6.02** ***	**10.4 ± 4.2** ***
**HbA1c (mmol/mol)**	31.24±12.6	**93.81 ± 18.17** ***	**93.31 ±25.50** ***
	5.0±3.3	**10.7±3.8** ***	**10.7±4.5** ***
**Systolic BP [mm Hg]**	108.7 ± 11.02	111.9 ± 15.7	**119 ± 15.98** **
**Diastolic BP [mmHg]**	69.95 ± 8.17	71.12 ± 7.57	**74.67 ± 6.12** *
**% Hypertensives**	Nil	3%	5%
**Total Cholesterol(mmol/L)**	9.38 ± 1.38	**8.7 ± 2.02** **	**9.0 ± 1.87** *
**HDL Cholesterol (mmol/L)**	2.71 ± 0.42	2.6 ± 0.65	2.86 ± 0.88
**LDL Cholesterol (mmol/L)**	6.08 ± 1.59	**5.1 ± 1.89** **	**5.08 ± 1.54** **
**Serum Triglycerides(mmol/L)**	4.41 ± 1.55	4.71 ± 1.91	4.79 ± 2.04
**GAD positivity [%]**	0	37	28
**Micro albuminuria (mg/dL)**	7.87 ± 12.28	8.98 ± 7.61	**97±107** ***
**Serum Urea (mmol/L)**	1.12 ± 0.25	1.38 ± 0.85	1.57 ±1.12*
**EPE(mg/day)**	103.4 ± 100.5	**124.9 ± 48.2** ***	**282 ± 397** ***
**Diabetic Retinopathy(DR) (%)**	0	0	42
**Diabetic Nephropathy(DN) (%)**	0	0	44
**DN + DR (%)**	0	0	14
**Disease duration [Years]**		8.23 ± 7.14	13.54 ± 6.57
**Treatment**	-	Insulin Anti-hypertensive drugs	Insulin Anti-hypertensive drugs

^a^- Comparison between NGT and T1DM;

^b^- Comparison between NGT and T1DM-MVC.

Data are given as mean± SD for continuos variables and as % for proportions. Statistical significance was determined by One-way ANOVA.

* p< 0.05;

**p< 0.01;

***p<0.0001.

FPG-fasting plasma glucose; HbA1C-glycated hemoglobin. Letters in bold highlight values which are significant.

**Fig 1:**
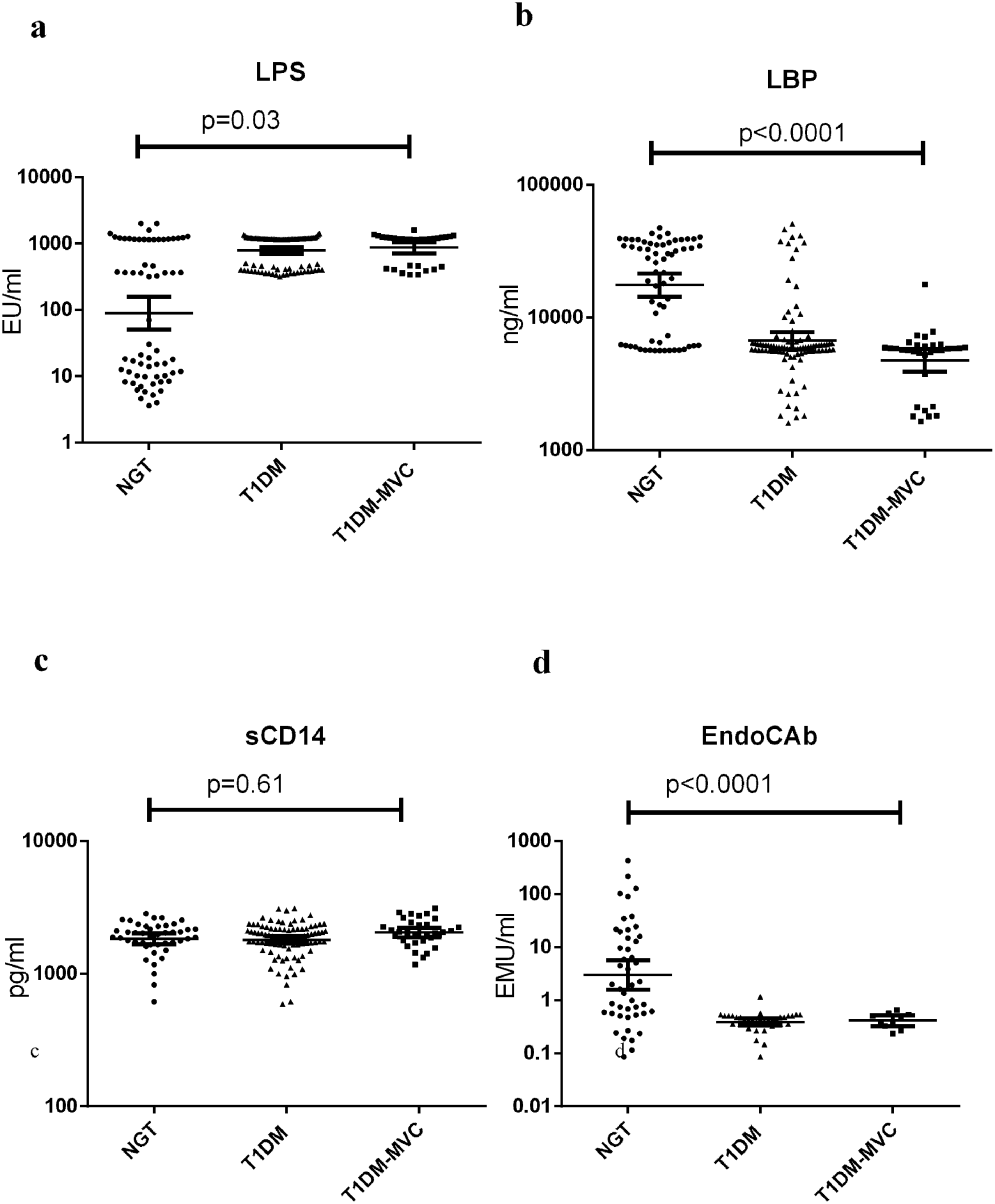
T1DM is characterized by systemic endotoxemia with significantly decreased levels of LBP and EndoCAb. Serum levels of LPS (a), LBP (b), sCD14 (c) and EndoCAb (d) were determined in NGT (n = 64), T1DM without MVC (n = 97) and T1DM subjects with MVC (n = 36) by immune-assays. Each dot represents individual values with the horizontal line representing the geo mean. 36 T1DM subjects without MVC and 26 with MVC had undetectable levels of EndoCAb. Significance was calculated by non-parametric Mann–Whitney U test and p<0.05 was considered significant. The indicated p values are for pair-wise comparisons.

Fig 1 shows the serum levels of LPS, LBP, sCD14 and EndoCAb in NGT and T1DM subjects (with and without MVC). Compared to NGT, T1DM subjects (both with and without MVC) had significantly higher levels of LPS, reduced levels of LBP and EndoCAb with no major difference in the sCD14 levels. T1DM subjects had undetectable levels of EndoCAb. No significant difference was seen in these biomarkers between T1DM subjects with and without MVC. At least among the NGT subjects (Fig 1a), two distinct sub-sects could be identified: one with high levels (MEAN 915 EU/ml) and another with low levels (MEAN 12 EU/ml) of LPS. On further analysis it was found that subjects who had high levels of endotoxemia also had high levels of IL-6, TNF-α and GM-CSF and low levels of LBP and EndoCAb. Interestingly this group also had significantly elevated levels of triglyceridemia (data not shown). Within the T1DM-MVC group, LPS, LBP, sCD14 and EndoCAb levels were not significantly different between those who had retinopathy from those who had microalbuminuria (LPS: DR(Mean ± SD)-1035±354 EU/ml, DN (Mean ± SD)- 943.8±441 EU/ml, p = 0.4; LBP:DR(Mean ± SD)-5± 1.8 μg/ml, DN(Mean ± SD)-6±3.9 μg/ml, p = 0.5; sCD14: DR(Mean ± SD)- 2.2±0.48 μg/ml and DN(Mean ± SD)-2.0±0.46 μg/ml, p = 0.2 and EndoCAb: DR(Mean ± SD)- 0.38±0.16 EMU/ml and DN(Mean ± SD)- 0.43±0.09 EMU/ml, p = 0.7).

**Fig 2:**
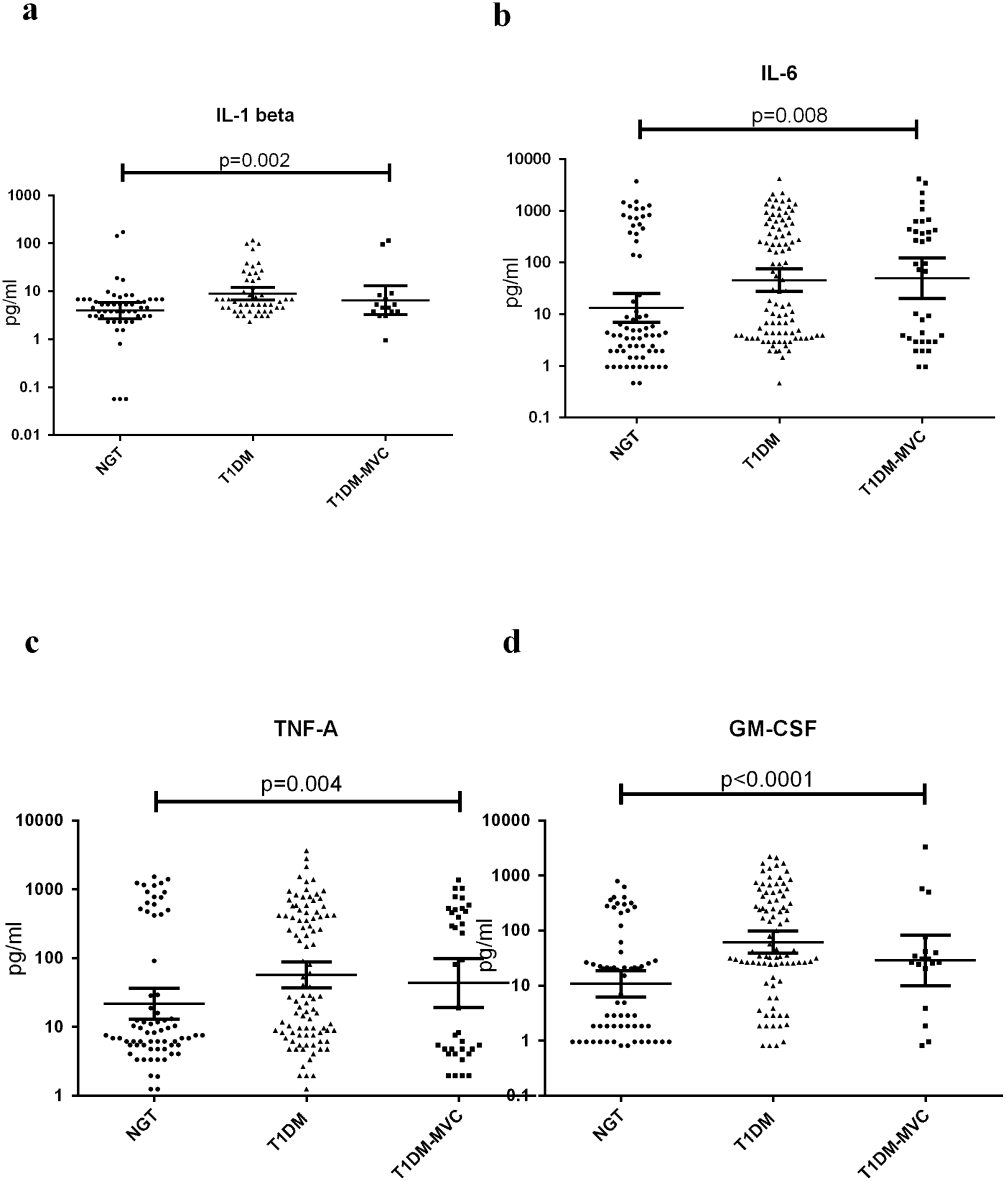
T1DM is characterized by systemic inflammation with high levels of serum pro-inflammatory cytokines. Serum levels of IL-1β (a), IL-6 (b), TNF-α (c), and GM-CSF (d) were determined in NGT (n = 64), T1DM without MVC (n = 97) and T1DM subjects with MVC (n = 36) by ELISA. Each dot represents individual values with the horizontal line representing the geo mean. Significance was calculated by non-parametric Mann–Whitney U test and p<0.05 was considered significant. The indicated p values are for pair-wise comparisons.

Fig 2 shows the serum levels of TNF-α, IL-6, IL-1β and GM-CSF in NGT and T1DM subjects (with and without MVC). Compared to NGT, TIDM subjects (both with and without MVC) had significantly elevated levels of TNF-α, IL-6, IL-1β and GM-CSF in the serum. No significant difference was seen in cytokine levels between T1DM subjects with and without MVC. Spearman’s correlation analysis within the diabetic group (S1 Table) showed positive correlation of LPS levels with FGP, EPE, IL-1β, IL-6, TNF-α and GM-CSF. LBP levels showed a negative correlation with BMI, FPG, SBP, DBP, TGL, EPE, IL-1β and IL-6 levels. EndoCAb showed negative correlation with FPG, EPE TNF-α and GM-CSF levels.

In the present study, we attempted to investigate LPS and its translocation markers in T1DM subjects with/without MVC by measuring serum LPS, EndoCAb, sCD14 and LBP along with serum cytokines and other clinical parameters. The major findings of the study are: 1. T1DM subjects with/without MVC had significantly higher levels of LPS and lower levels of LBP and EndoCAb with no significant change in the sCD14 levels compared to NGT. 2. However, even for these markers, there was no significant difference between T1DM subjects with and without MVC and 3. While LPS showed a positive correlation FPG and pro-inflammatory cytokines (IL-1β, IL-6 and TNF-α), LBP and EndoCAb showed negative association with these markers.

Changes in gut microbiota leading to leaky gut have now been identified as a major etiological factor for chronic inflammation as seen in T1DM [14]. Previously, increased levels of serum Zonulin, a gap-junction protein which serves as a marker for gut permeability was found to be significantly elevated in T1DM subjects indicating leaky gut [15]. However, comparatively very few studies have looked at the end result of leaky gut which is the metabolic endotoxemia and associated inflammation. Recently, increased levels of serum LPS in T1DM subjects with metabolic syndrome and/or kidney disease was reported [16, 17]. However, the levels of sCD14, LBP and EndoCAb which are the major regulators of LPS activity under *in vivo* conditions were not reported in these subjects. LBP is an acute-phase protein that enhances the LPS responsiveness of immune cells by transferring LPS to CD14 [18]. Even though an acute-phase protein, LPB levels were not significantly elevated in T1DM subjects. Previously, we have reported metabolic endotoxemia with no significant change in serum LBP levels in coronary artery disease (CAD) patients [19], which is a classical chronic inflammatory condition. However, others have reported significantly elevated LBP levels under conditions of chronic inflammation like obesity [20], Type-2 diabetes [21] and non-alcoholic fatty-liver disease [22]. The disparity could be due to the biphasic role of LBP in LPS induced chronic inflammation: At lower concentrations it can actually fuel inflammation while at higher concentrations it tends to neutralize LPS activity and promotes its degradation [23]. High levels of LBP inhibit the LPS responses in monocytes and can protect both humans [24] and mice [25] from septic shock caused by LPS or gram-negative bacterial infection. The low level of LBP seen in the T1DM subjects suggests increased activity of LPS in these subjects. Low levels of LBP has also been reported under conditions of extra-pulmonary tuberculosis [26] and active filariasis [27]. However, this is the first report, to the best of our knowledge, to show low LBP levels in autoimmunity. Serum EndoCAb levels serve as good prognostic marker for post-operative mortality rate associated with acute inflammation as seen in septicemia and endotoxemia [28]. But the significance of EndoCAb levels under conditions of autoimmunity is less well explored. Our study for the first time showed low levels of EndoCAb in T1DM subjects.

The moderate endotoxemia with decreased EndoCAb and LBP together suggests sustained immune activation resulting in chronic inflammation. The end result is a systemic increase in the levels of IL-1β, TNF-α, IL-6 and GM-CSF, as seen in this study. Several immune and non-immune cells express LPS receptor (TLR4) and respond to LPS stimulation by secreting pro-inflammatory cytokines [29]. The end result is a systemic increase in the levels of IL-1β, TNF-α, IL-6 and GM-CSF in the T1DM subjects as seen in this study. Our results are in agreement with previous reports were in elevated levels of IL-1β and IL-6 were reported in T1DM subjects [30]. Recently we reported increased levels of serum GM-CSF levels in T2DM subjects which were associated with the activated phenotype of dendritic cells [31]. Like T2DM, GM-CSF levels were found to be elevated even in T1DM subjects as can be seen in this study.

## Conclusion

To summarize, chronic low-grade endotoxemia, as seen in the case of T1DM, was associated with decreased levels of LBP and EndoCAb and with elevated levels of TNF-α, IL-6, IL-1β and GM-CSF, indicating chronic inflammation. The most important finding of this study is that, this phenomenon was seen even during the early stages of the disease and seems to persist till the late stages of the disease were in MVC sets in, indicating that, yet to be identified factors may play a role in the onset of MVC in T1DM subjects. One of the limitations of this study is that, being cross-sectional in nature, no conclusions regarding a causal relationship between autoimmunity, endotoxemia and inflammation can be made. Further, since the prevalence of T1DM is less than 1% [32] this study was performed in a limited sample size with known diabetic subjects. However, the strength of this study is that, it systematically reviews all the components of microbial translocation in age and gender matched T1DM subjects in a non-Caucasians population were in the incidence of T1DM has started increasing. In summary, this study suggests that T1DM in Asian Indians is characterized by high levels of LPS and pro-inflammatory cytokines and low levels of EndoCAb and LBP.

## Supporting Information

S1 Table Spearman’s Correlation analysis for LPS and translocation markers with clinical parameters and log cytokine concentration within the diabetic group (n = 133). (DOC)

## Abbreviations

T1DM: Type-1 diabetes mellitus
MVC: microvascular complication
NGT: Normal glucose tolerance
LPS: Lipopolysaccharide
LBP: Lipopolysaccharide binding protein
sCD14: Soluble cluster differentiation 14
EndoCAb: Endotoxin core antibody
TNF-α: Tumor necrosis factor- alpha
IL-1β: Interleukin-1 beta
GM-CSF: Granulocyte macrophage colony stimulating factor
IL-6: Interleukin-6
FPG: Fasting Plasma Glucose
SBP: Systolic Blood Pressure
DBP: Diastolic Blood Pressure
LDL: Low Density Lipoprotein
HDL: High Density Lipoprotein
TGL: Triglyceride Lipids
DN: Diabetic Nephropathy
DR: Diabetic Retinopathy
EPE: Expected Protein Excretion
GAD: Glutamate decarboxylase
