# Supplementary material for: Chronic Endotoxemia in Subjects with Type-1 Diabetes Is Seen Much before the Onset of Microvascular Complications
Source: PLoS One. 2015 Sep 14;10(9):e0137618. doi: 10.1371/journal.pone.0137618 (PMC4569180; doi:10.1371/journal.pone.0137618)
Supplement: S1 Table — (DOC) [file pone.0137618.s001.doc]

**S.Table-2: Spearman’s Correlation analysis for LPS and translocation markers with clinical parameters and log cytokine concentration within the diabetic group (n=133)**

|  | LPS | |  | LBP | |  | sCD14 | |  | EndoCAb | |
| --- | --- | --- | --- | --- | --- | --- | --- | --- | --- | --- | --- |
|  | r | p |  | r | p |  | r | p |  | r | p |
| Age | 0.051 | 0.654 |  | -0.199 | 0.078 |  | 0.09 | 0.416 |  | 0.01 | 0.933 |
| BMI | 0.102 | 0.370 |  | **-0.298** | **0.008^**^** |  | 0.102 | 0.372 |  | -0.061 | 0.596 |
| Systolic BP | 0.162 | 0.153 |  | **-0.335** | **0.003^**^** |  | 0.081 | 0.480 |  | -0.182 | 0.109 |
| Diastolic BP | 0.099 | 0.385 |  | **-0.287** | **0.010^**^** |  | -0.042 | 0.713 |  | -0.190 | 0.094 |
| FPG | **0.487** | **<0.001^***^** |  | **-0.475** | **<0.001^***^** |  | -0.111 | 0.342 |  | **-0.500** | **<0.001^***^** |
| TGL | 0.197 | 0.096 |  | **-0.247** | **0.036^*^** |  | -0.089 | 0.458 |  | -0.299 | 0.011^**^ |
| HDL | -0.038 | 0.749 |  | 0.107 | 0.369 |  | 0.108 | 0.366 |  | 0.033 | 0.785 |
| LDL | -0.182 | 0.126 |  | 0.174 | 0.144 |  | -0.166 | 0.165 |  | 0.229 | 0.053 |
| Cholesterol | -0.131 | 0.211 |  | 0.159 | 0.180 |  | -0.167 | 0.159 |  | 0.147 | 0.216 |
| Urea | 0.152 | 0.182 |  | **-0.265** | **0.018^**^** |  | -0.094 | 0.408 |  | -0.157 | 0.168 |
| Creatinine | 0.118 | 0.299 |  | -0.210 | 0.063 |  | 0.066 | 0.565 |  | -0.186 | 0.100 |
| EPE | **0.363** | **0.01^**^** |  | -0.413 | 0.003^**^ |  | -0.127 | 0.384 |  | **-0.422** | **0.003^**^** |
| Microalbuminuria | 0.078 | 0.51 |  | -0.207 | 0.079 |  | -0.105 | 0.37 |  | -0.147 | 0.215 |
| IL-1 β | **0.428** | **<0.001^***^** |  | -0.219 | 0.068 |  | -0.017 | 0.890 |  | -0.218 | 0.069 |
| IL-6 | **0.245** | **0.041^*^** |  | **-0.323** | **0.006^**^** |  | -0.028 | 0.818 |  | -0.139 | 0.252 |
| TNF-α | **0.312** | **0.009^**^** |  | -0.147 | 0.224 |  | -0.162 | 0.179 |  | **-0.338** | **0.004^**^** |
| GM-CSF | **0.343** | **0.009^**^** |  | **-0.391** | **0.003^**^** |  | -0.063 | 0.644 |  | **-0.288** | **0.030^*^** |
|  |  |  |  |  |  |  |  |  |  |  |  |

* p < 0.05; **p< 0.01; ***<0.001.

LPS, lipopolysaccharide; LBP-lipopolysaccharide binding protein; sCD14, soluble CD14; EndoCAb, endotoxin core antibody; BMI,body mass indexl FPG, fasting plasma glucose; BP, blood pressure; HDL, high-density lipoprotein;LDL,low density lipoprotein;EPE,expected protein excretion. Letters in bold highlight r values which are significant.
